# Supplementary material for: ADAM17 targeting by human cytomegalovirus remodels the cell surface proteome to simultaneously regulate multiple immune pathways
Source: Proc Natl Acad Sci U S A. 2023 Aug 10;120(33):e2303155120. doi: 10.1073/pnas.2303155120 (PMC10438378; doi:10.1073/pnas.2303155120)
Supplement: Supplementary file 1 — Appendix 01 (PDF) [file pnas.2303155120.sapp.pdf]

Supplemental Figure 1

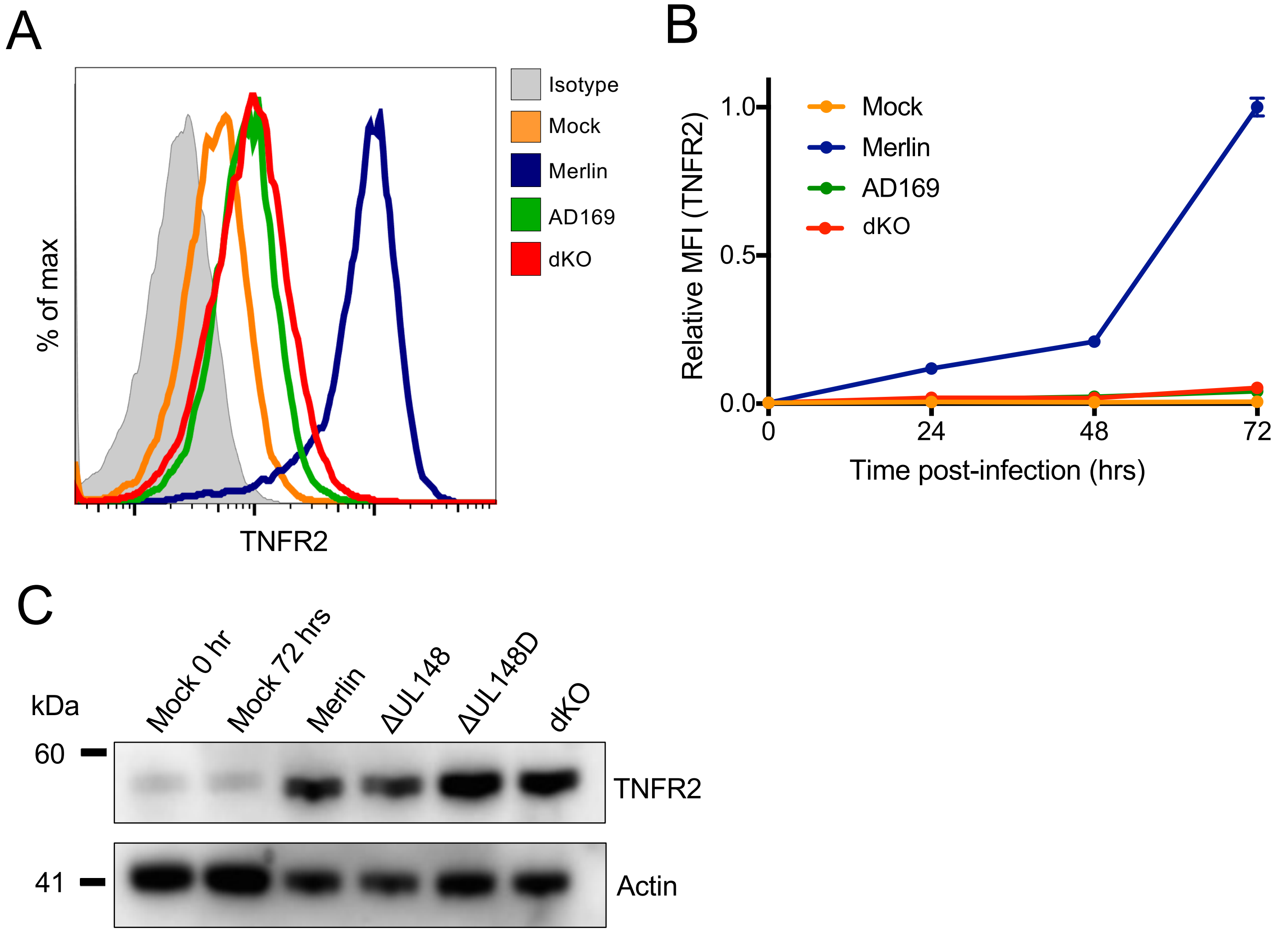

Action of UL148 and UL148D individually and together on expression of TNFR2. HF-TERT cells were mock-infected or infected with the indicated HCMV strains and analyzed by flow cytometry at 72 hrs pi (A) or at indicated time points pi (B) for surface expression of TNFR2. MFI values are shown relative to Merlin-infected cells (set to 1) at 72 hrs pi. (C) HF-TERT cells were mock-infected or infected with HCMV strain Merlin or the indicated deletion mutants, and whole-cell lysates were analyzed by immunoblotting at 72 hrs pi for TNFR2. Actin was used as a loading control.

Supplemental Figure 2

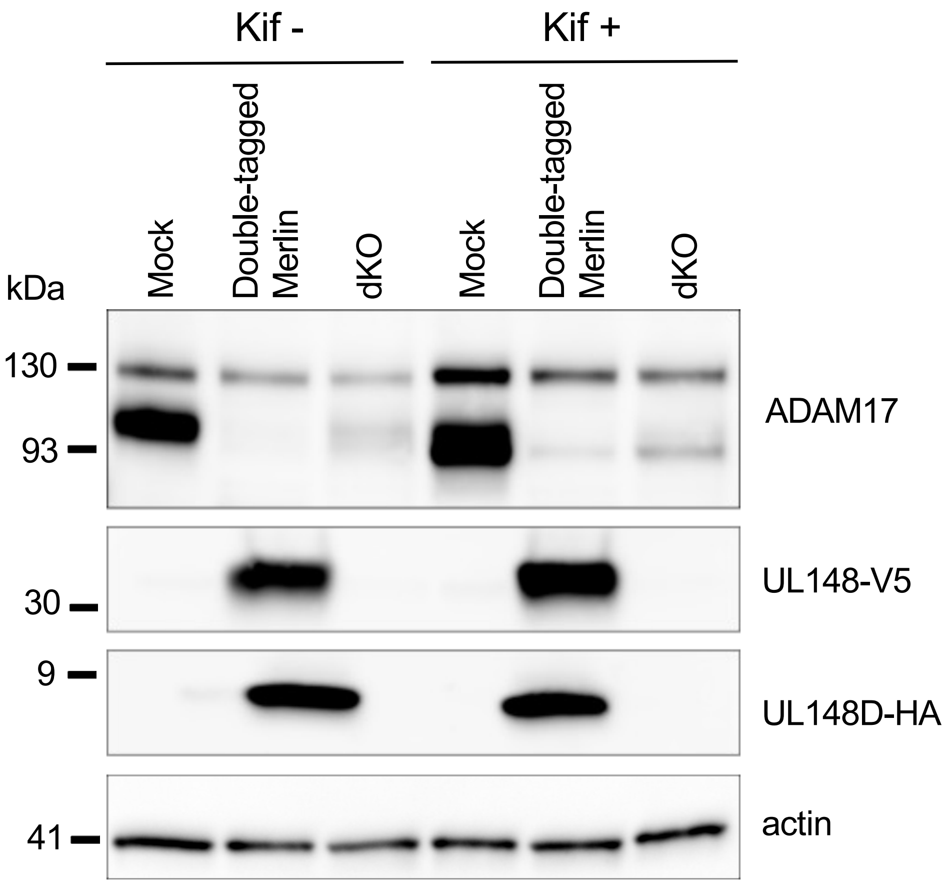

Kifunensine treatment does not recover mature ADAM17 expression in Merlin-infected cells to the levels observed in dKO-infected cells. HF-TERTs were uninfected (Mock), or infected with the indicated HCMV strains following the addition of 2.5 μM Kifunensine (Kif) or water. Immunoblotting of ConA enriched ADAM17 in the presence or absence of Kif at 72 hpi with HCMVs (MOI = 10) and samples processed at 96 hpi. UL148 was tagged with V5 (UL148-V5) and UL148D with HA (UL148D-HA). Actin shown as a control for loading.

Supplemental Figure 3

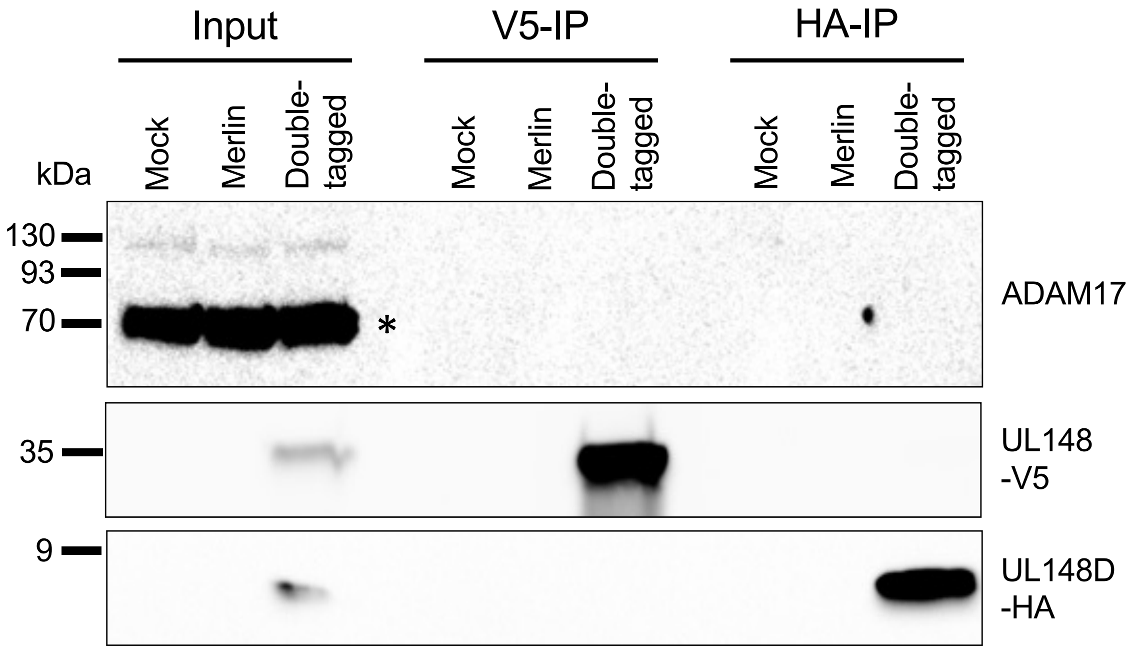

Immunoprecipitation with UL148-V5 or UL148D-HA tags and Western blotting for ADAM17 during HCMV infection. HF-TERTs were infected with Mock, Merlin or Double-tagged (UL148-V5, UL148D-HA) HCMV on a Merlin background (72 hpi, MOI = 10). At 72 hpi cells were lysed and IP performed using either V5- or HA-tag agarose. Samples were ran on SDS-PAGE, followed by a transfer to a PVDF membrane and staining for ADAM17, V5- and HA- tags. *ADAM17 Western blotting without ConA enrichment results in a highly abundant band at 70 kDa, while the glycosylated protein signals are weak relative to this signal. Methodology is as described (Kunzel *et al*. (2018) FRMD8 promotes inflammatory and growth factor signalling by stabilising the iRhom/ADAM17 sheddase complex. *eLife*, 7: e35012). ConA enrichment cannot be performed on lysates in conjunction with immunoprecipitation due to intrinsic technical differences between the protocols (Dusterhoft *et al*. (2021) The iRhom homology domain is indespensible for ADAM17-mediated TNFα and EGF receptor ligand release. *Cell Mol Life Sci*, 78: 5015-40).
